# The urgent need for pan-antiviral agents: from multitarget discovery to multiscale design

**DOI:** 10.4155/fmc-2020-0134

**Published:** 2020-11-23

**Authors:** Valeria V Kleandrova, Alejandro Speck-Planche

**Affiliations:** ^1^Laboratory of Fundamental & Applied Research of Quality & Technology of Food Production, Moscow State University of Food Production, Volokolamskoe shosse 11, PO Box 125080, Moscow, Russian Federation; ^2^Programa Institucional de Fomento a la Investigación, Desarrollo e Innovación, Universidad Tecnológica Metropolitana, Ignacio Valdivieso 2409, PO Box 8940577, San Joaquín, Santiago, Chile

**Keywords:** COVID-19 • drug repurposing, multitarget inhibitor, multiscale *de novo* drug design, new molecular entity, pan-antiviral, PTML, SARS-CoV-2

Viral infections continue to plague mankind. In the last 20 years the world has witnessed the increased emergence of different viral outbreaks: SARS in 2002, influenza A (H1N1) in 2009, the Middle East respiratory syndrome in 2012, Western/Eastern equine encephalitis in 2013 and Ebola in 2014. All these viral diseases are lethal and highly transmissible through travelers [1]. For most of these viruses, there are no treatments able to eradicate them. Recently, SARS-CoV-2, responsible for the COVID-19 pandemic has emerged, leaving a trail of more than 32.5 million infected people and more than 989,000 deaths as of 26 September 2020 [2]; the great morbidity and mortality associated with SARS-CoV-2/COVID-19 are yet expected to increase. The period between these outbreaks has shortened, suggesting that more viral epidemics will be seen in the years to come. Consequently, there is an urgent need to discover or design pan-antiviral agents (also known as broad-spectrum antivirals). In this article we highlight the different aspects that point to the development of pan-antiviral agents and the most promising *in silico* methodologies that can expedite this process. Emphasis is placed on finding potential treatments against SARS-CoV-2/COVID-19 from the perspective of discovering pan-antivirals which could also assist with the elimination of other viral pathogens.

## The decadence of the one-target-one-drug paradigm

The vast majority of the approaches at both computational and experimental levels have focused on discovering drugs that inhibit specific targets. However, the many success stories in developing drugs to treat diseases barely compensate for the remarkably high expenditure of time (at least 12 years) and financial resources (around US$3 billion per new drug approved) [3]. Thus, the one-drug-to-one-target paradigm in drug discovery is characterized by many failures and great uncertainty. The most recent example demonstrating the drawbacks of designing highly specific monotargeted drugs is the disappointing result obtained in preliminary clinical trials by attempting to repurpose the drug combination lopinavir/ritonavir to treat SARS-CoV-2 [4]. These are well-known anti-HIV drugs acting as protease inhibitors, which were expected to have *in vivo* efficacy against SARS-CoV-2 by inhibiting the viral protein 3CLpro. The negative result of this study suggests two possible options: either antivirals should eradicate SARS-CoV-2 by inhibiting targets related to other processes, such as entry or replication, or they should act as multitarget inhibitors, disrupting several pathways necessary for virus survival and/or infectivity. Regarding the first option, DrugBank [5] reports 42 of the most promising anti-SARS-CoV2 experimental treatments [6]. These therapies focus on vaccines (for example, mRNA-1273 and Ad5-nCoV), antibodies (bevacizumab, leronlimab and others), convalescent plasma or different US FDA-approved (or experimental) drugs for medical conditions other than COVID-19; the latter group includes (but is not limited to) inhibitors of viral proteins such as protease (darunavir, GC-373, etc.) and RdRp (favipiravir and nucleoside analogs [remdesivir and galidesivir]), as well as blockers of ACE2 glycosylation (chloroquine) and endosomal acidification (hydroxychloroquine), just to cite a few examples. Although these treatments may prove effective against SARS-CoV-2/COVID-19, their use as pan-antivirals is limited because they exhibit highly specific mechanisms of action. For instance, favipiravir and remdesivir are two pan-antiviral drugs that act as selective RdRp inhibitors across different viruses. Nevertheless, RdRp-exonuclease interactions imbue coronaviruses with different degrees of resistance to RdRp inhibitors [7], thus limiting the potential applications of these antiviral agents. To the best of our knowledge, the second option – which focuses on searching for pan-antivirals acting as multitarget inhibitors – has not been explored yet. We would like to stress that multitarget drug discovery is a more complete paradigm than its one-drug-to-one target counterpart because finding multitarget inhibitors will also reveal information about target preferences and selectivity, thus providing insights for the design of selective inhibitors.

## Drug repurposing of FDA-approved drugs as pan-antiviral agents using advanced computational models

In the race to find potential treatments against SARS-CoV-2/COVID-19 and other viruses, *in silico* models could serve as tools to identify FDA-approved drugs that can behave as pan-antivirals. Popular techniques relying on structure-based approaches (e.g., molecular docking) will always be useful but are computationally demanding if applied to the search for pan-antivirals via multitarget inhibition against diverse viral proteins. Therefore we will focus on 2D ligand-based approaches, which can be performed faster and without the diminution of accuracy. We would like to highlight three popular/important approaches that can be used to repurpose FDA-approved drugs as pan-antiviral agents. Each of them either directly or indirectly benefits from the fact that some proteins remain conserved across different viral species. Thus even if there is little information about an emerging pathogen, inhibiting different proteins of already known viruses (or the viruses themselves) could provide a starting point for inhibiting the new virus.

The first approach focuses on models combining perturbation theory and machine learning (PTML) [8,9] which, through the application of Box–Jenkins operators, can integrate *in vitro* screening data containing multiple viral proteins from different viruses. This enables the screening of FDA-approved drugs, and those predicted as versatile inhibitors of the viral proteins can be experimentally validated as potential treatments against SARS-CoV-2 and other viral pathogens. In the second approach, PTML models are applied to virtually screen drugs against different viruses in inhibition/replication assays. Again, the drugs predicted as pan-antivirals will be prioritized in biological testing against SARS-CoV-2. We would like to emphasize that, in antiviral research, PTML models have found successful applications in modeling anti-HIV activity through multitarget inhibition [10], linking *in vitro* activity with epidemiological outcomes [11] and virtually screening antiretroviral compounds [12]. Therefore PTML models could be easily adapted and built for the two aforementioned cases.

The third approach involves the development of alignment-free multitarget (AFMT) models [13]. These computational tools are constructed by calculating molecular descriptors from both the 2D structures of the ligands/inhibitors and the proteins’ amino acid sequences. The advantage of the AFMT models over their PTML counterparts is that the former can predict the inhibitory potency of large libraries of (either virtual or already synthesized) chemicals against proteins other than those used to develop the AFMT models [13,14]. AFMT models can therefore screen FDA-approved drugs against SARS-CoV-2 proteins (including the spike protein), whose sequences can be retrieved from public sources such as UniProt [15] or Protein Data Bank [16]; the drugs predicted as multitarget inhibitors of the SARS-CoV-2 proteins can then be fast-tracked for experimental confirmation.

## Designing new molecular entities as potent & safe pan-antivirals

Considering the accumulated experience, the multifactorial nature of viral infections and the limitations of current antiviral therapies, new molecular entities (NMEs) could be computationally designed as pan-antivirals. We are referring to a chemical able to inhibit multiple viruses that are dissimilar in terms of their genomes, replication, transmission, mutability and infection mechanisms. Such a pan-antiviral agent will offer a better chance of eliminating SARS-CoV-2 and other viral pathogens. To reduce both the response time against a viral pandemic and financial resources, the chosen computational approach must integrate both *in vitro* and *in vivo* assays while focusing on designing a small library of 5–20 chemicals that virtually exhibit potent pan-antiviral activity, low toxicity and adequate pharmacokinetic properties.

To date, the most suitable approach capable of designing an NME is multiscale *de novo* drug design [17], which involves four steps. First, a multitasking model for quantitative structure–biological effect relationships (mtk-QSBER) is built [18]. Then the most common molecular fragments present in the dataset used to create the model are extracted and their quantitative contributions to the pan-antiviral activity and the safety profiles are calculated; fragments with positive contributions will be used as building blocks to design NMEs. Third, the molecular descriptors present in the mtk-QSBER model are interpreted, providing information regarding the physicochemical properties and structural features that a molecule should possess to simultaneously display pan-antiviral activity, reduced toxicity and desirable pharmacokinetic profiles. Last, NMEs are generated by assembling fragments with positive contributions according to the interpretations of the molecular descriptors. In the context of antiviral research, multiscale *de novo* drug design has been successfully applied to the virtual generation of anti-hepatitis C agents [19] and anti-HIV compounds [20]. This approach represents the fastest and most economical solution to discover potent and safe pan-antivirals while preventing promiscuous (off-target) interactions potentially associated with side effects.

## Conclusion

Viral infections represent a constant threat to humanity. The latest viral outbreaks have derived from RNA-based viruses, but it must not be taken for granted that DNA-based viruses are unable to cause pandemics. Developing pan-antiviral agents is perhaps the most efficient way to tackle all the emerging and re-emerging viral pathogens. The search for such therapeutic agents should be performed with the aim of finding chemicals that will disrupt dissimilar mechanisms of action, stopping the biomolecular machinery of viral infection and/or replication across multiple viral species. Computational approaches are and will be essential in accelerating the search for pan-antiviral agents, either by repurposing drugs with multitarget inhibition patterns via the PTML and AFMT models, or by using multiscale *de novo* drug design to rationally generate NMEs with the desired properties.

## References

[1] Zhu J-D, Meng W, Wang X-J, Wang H-CR Broad-spectrum antiviral agents. Front. Microbiol. 6, 517 (2015). 26052325 10.3389/fmicb.2015.00517 PMC4440912

[2] Dong E, Du H, Gardner L An interactive web-based dashboard to track COVID-19 in real time. Lancet Infect. Dis. 20(5), 533–534 (2020). 32087114 10.1016/S1473-3099(20)30120-1 PMC7159018

[3] DiMasi JA, Grabowski HG, Hansen RW Innovation in the pharmaceutical industry: new estimates of R&D costs. J. Health Econ. 47, 20–33 (2016). 26928437 10.1016/j.jhealeco.2016.01.012

[4] Dong L, Hu S, Gao J Discovering drugs to treat coronavirus disease 2019 (COVID-19). Drug Discov. Ther. 14(1), 58–60 (2020). 32147628 10.5582/ddt.2020.01012

[5] Wishart DS, Knox C, Guo AC DrugBank: a comprehensive resource for *in silico* drug discovery and exploration. Nucleic Acids Res. 34(Database issue), D668–672 (2006). 16381955 10.1093/nar/gkj067 PMC1347430

[6] DrugBank: COVID-19 information (2020). https://go.drugbank.com/covid-19

[7] Shannon A, Le NT-T, Selisko B Remdesivir and SARS-CoV-2: structural requirements at both nsp12 RdRp and nsp14 Exonuclease active-sites. Antiviral Res. 178, 104793 (2020). 32283108 10.1016/j.antiviral.2020.104793 PMC7151495

[8] Nocedo-Mena D, Cornelio C, Camacho-Corona MDR Modeling antibacterial activity with machine learning and fusion of chemical structure information with microorganism metabolic networks. J. Chem. Inf. Model. 59(3), 1109–1120 (2019). 30802402 10.1021/acs.jcim.9b00034

[9] Kleandrova VV, Ruso JM, Speck-Planche A, Dias Soeiro Cordeiro MN Enabling the discovery and virtual screening of potent and safe antimicrobial peptides. Simultaneous prediction of antibacterial activity and cytotoxicity. ACS Comb. Sci. 18(8), 490–498 (2016). 27280735 10.1021/acscombsci.6b00063

[10] Speck-Planche A, Kleandrova VV, Luan F, Cordeiro MNDS A ligand-based approach for the *in silico* discovery of multi-target inhibitors for proteins associated with HIV infection. Mol. Biosyst. 8(8), 2188–2196 (2012). 22688327 10.1039/c2mb25093d

[11] Gonzalez-Diaz H, Herrera-Ibata DM, Duardo-Sanchez A, Munteanu CR, Orbegozo-Medina RA, Pazos A ANN multiscale model of anti-HIV drugs activity vs AIDS prevalence in the US at county level based on information indices of molecular graphs and social networks. J. Chem. Inf. Model. 54(3), 744–755 (2014). 24521170 10.1021/ci400716y

[12] Vasquez-Dominguez E, Armijos-Jaramillo VD, Tejera E, Gonzalez-Diaz H Multioutput perturbation-theory machine learning (PTML) model of ChEMBL data for antiretroviral compounds. Mol. Pharm. 16(10), 4200–4212 (2019). 31426639 10.1021/acs.molpharmaceut.9b00538

[13] Speck-Planche A, Kleandrova VV, Scotti MT, Cordeiro MNDS 3D-QSAR methodologies and molecular modeling in bioinformatics for the search of novel anti-HIV therapies: rational design of entry inhibitors. Curr. Bioinform. 8(4), 452–464 (2013).

[14] Vina D, Uriarte E, Orallo F, Gonzalez-Diaz H Alignment-free prediction of a drug-target complex network based on parameters of drug connectivity and protein sequence of receptors. Mol. Pharm. 6(3), 825–835 (2009). 19281186 10.1021/mp800102c

[15] Apweiler R, Bairoch A, Wu CH UniProt: the universal protein knowledgebase. Nucleic Acids Res. 32(Database issue), D115–119 (2004). 14681372 10.1093/nar/gkh131 PMC308865

[16] Berman HM, Westbrook J, Feng Z The Protein Data Bank. Nucleic Acids Res. 28(1), 235–242 (2000). 10592235 10.1093/nar/28.1.235 PMC102472

[17] Speck-Planche A Recent advances in fragment-based computational drug design: tackling simultaneous targets/biological effects. Future Med. Chem. 10(17), 2021–2024 (2018). 30058835 10.4155/fmc-2018-0213

[18] Speck-Planche A, Cordeiro MNDS Multitasking models for quantitative structure-biological effect relationships: current status and future perspectives to speed up drug discovery. Expert Opin. Drug Discov. 10(3), 245–256 (2015). 25613725 10.1517/17460441.2015.1006195

[19] Speck-Planche A, Cordeiro MNDS Speeding up early drug discovery in antiviral research: a fragment-based *in silico* approach for the design of virtual anti-hepatitis C leads. ACS Comb. Sci. 19(8), 501–512 (2017). 28437091 10.1021/acscombsci.7b00039

[20] Kleandrova VV Multitasking model for computer-aided design and virtual screening of compounds with high anti-HIV activity and desirable ADMET properties. : Multi-Scale Approaches in Drug Discovery. Speck-Planche A (Ed.). Elsevier, 55–81 (2017).

